# Characterization and classification of adherent cells in monolayer culture using automated tracking and evolutionary algorithms

**DOI:** 10.1016/j.biosystems.2016.05.009

**Published:** 2016-08

**Authors:** Zhen Zhang, Matthew Bedder, Stephen L. Smith, Dawn Walker, Saqib Shabir, Jennifer Southgate

**Affiliations:** a Department of Electronics, University of York, Heslington, York YO10 5DD, UK; b Department of Computer Science, University of York, Heslington, York YO10 5GW, UK; c Department of Computer Science & Insigneo, Institute for in silico Medicine, University of Sheffield, Sheffield S1 4DP, UK; d Jack Birch Unit, Department of Biology, University of York, Heslington, York YO10 5DD, UK

## Abstract

This paper presents a novel method for tracking and characterizing adherent cells in monolayer culture. A system of cell tracking employing computer vision techniques was applied to time-lapse videos of replicate normal human uro-epithelial cell cultures exposed to different concentrations of adenosine triphosphate (ATP) and a selective purinergic P2X antagonist (PPADS), acquired over a 24 h period. Subsequent analysis following feature extraction demonstrated the ability of the technique to successfully separate the modulated classes of cell using evolutionary algorithms. Specifically, a Cartesian Genetic Program (CGP) network was evolved that identified average migration speed, in-contact angular velocity, cohesivity and average cell clump size as the principal features contributing to the separation. Our approach not only provides non-biased and parsimonious insight into modulated class behaviours, but can be extracted as mathematical formulae for the parameterization of computational models.

## 1 Introduction

Urothelium is a remarkable epithelial tissue that lines the bladder and associated urinary tracts forming the tightest and most efficient self-repairing barrier in the body. In response to physical or other damage, the urothelium switches rapidly and transiently from a stable, mitotically-quiescent barrier into a highly proliferative state. The mechanisms that facilitate this switch are central to the pathophysiology of the bladder, but are poorly understood.

The urothelium is reported to respond to mechanical and chemical stimulation by releasing soluble factors, including adenosine triphosphate (ATP), which are proposed to play a role in mediating neuronal signalling (Birder, 2011). In addition, the urothelium expresses purinergic P2X and P2Y receptors and channels that are responsive to ATP released from autocrine or paracrine sources (Shabir et al., 2013). The outcome of such signalling is incompletely understood, as it could have a feedback role in modulating neuronal signalling, but alternatively could play a more direct role in urothelial barrier repair (Shabir et al., 2013). It has been further suggested that aberrant expression of receptors and/or mediator release by the urothelium is involved in dysfunctional diseases of the bladder, including idiopathic detrusor instability and interstitial cystitis (Birder and de Groat, 2007).

Despite the reported expression of these channels and receptors by the urothelium, consensus has been confounded by inconsistencies in experimental approaches, including the species, specificity of reagents, and the nature of the tissue preparation (reviewed (Yu and Hill, 2011)). We have developed a cell culture system for investigating normal human urothelial (NHU) cells and tissues in vitro. In previous work using this culture system, we showed that stimulation of P2 receptors with exogenous ATP enhanced scratch wound repair, as did addition of the ecto-ATPase inhibitor ARL-67156, which prevents the breakdown of autocrine-produced ATP. By contrast, blockade of P2X activity inhibited scratch wound repair in either the presence or absence of ATP (Shabir et al., 2013). This indicates that ATP is one of the major factors released upon urothelial damage and that it is likely to contribute to urothelial barrier repair.

To understand further the effect of ATP and P2X signalling on urothelial cell phenotype, time-lapse videos have been generated of low density urothelial cell cultures to which exogenous ATP and selective antagonists of P2X have been applied. This paper describes the development of an automated method for objective measurement of these videos using computer vision techniques, followed by the extraction of features, with the aim of identifying key characteristics of cell behaviour related to differences in the population. Replicate cell cultures are prepared in parallel and recorded over a 24-h period using standard videomicroscopy. The digital videos are then processed using custom cell tracking software implemented using a range of computer vision techniques. The resulting tracking data is then subjected to two methods of analysis with the aim of characterizing the behaviour of the cell cultures. The first is the extraction of a set of features informed from previous research and specified by the biological motivation for this study. The second approach is the application of a novel classifier employing *evolutionary algorithms* − computer programs whose operation is inspired by the processes of Darwinian evolution. These algorithms have the potential to provide power classifiers, as well as revealing those biological properties that contribute to the classification.

Section 2 of this paper describes the underlying biological processes of the urothelium in greater depth and then provides an overview of current modelling, along with an introduction to evolutionary algorithms. The processes and methodology adopted in our work are described in Section 3, and results, with statistical analysis, are presented in Section 4. Finally, conclusions and future work are considered in Section 5.

## 2 Background

### 2.1 The urothelium – a relevant tissue-specific experimental cell system

Urothelium, the transitional epithelium found lining the bladder and associated urinary tract functions as a stable, but self-repairing urinary barrier. This barrier function is attributable to urothelium-specific specialisations acquired during differentiation of the superficial cells, with surface membrane plaques composed of uroplakins (Hu et al., 2002) and well-developed intercellular tight junctions (Smith et al., 2015) Within the urothelium, individual cells are maintained in a mitotically-quiescent state until, following damage to the barrier, cells from all layers switch to a proliferative phenotype in order to effect efficient barrier repair (Varley et al., 2005). The balance between the paradoxical processes of regeneration and differentiation is critical to maintaining an effective urinary barrier, but the mechanisms that regulate urothelial tissue homeostasis and the switch between quiescent and regenerative (self-repair) phenotypes are poorly understood.

**Fig. 1:**
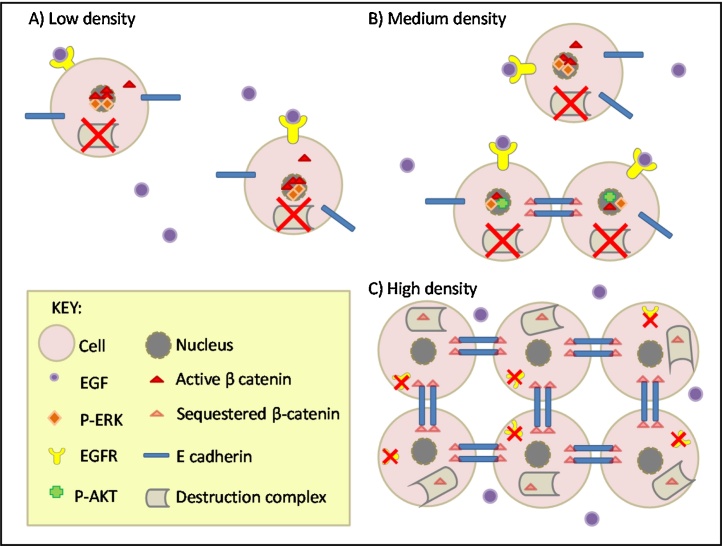
Schematic model of Wnt/β-catenin signalling crosstalk with EGFR/ERK and cell:cell contact-mediated β-catenin regulation in NHU cell proliferation.

Reproduced with permission from (Georgopoulos et al., 2014).

We have developed a robust and experimentally-tractable culture system for isolated normal human urothelial (NHU) cells in which regenerative and functionally-differentiated barrier states can be replicated (Baker et al., 2014). In the simplest case, NHU cells maintained in low calcium, serum-free conditions adopt a “basal” epithelial cell phenotype, in which cells grow as non-stratified, highly proliferative and migratory cultures that become contact-inhibited at confluence (Southgate et al., 1994). By modifying E-cadherin (adherens) contacts (by switching exogenous calcium from low [0.09 mM] to near-physiological [2 mM], or by retroviral transduction of a dominant negative E-cadherin (H-2K^d^-E-cad) construct), we have shown that the stability of E-cadherin cell–cell contacts is responsible for differentially regulating population growth through the Epidermal Growth Factor Receptor (EGFR)/Extracellular Signal-Regulated Kinase (ERK) and Phosphatidylinositol 3-Kinase (PI3-K)/AKT signalling pathways (Georgopoulos et al., 2010). Stable adherens junctions down-regulate the EGFR/ERK pathway, whilst inducing PI3-K/AKT activity to promote proliferation at low cell density. Functional inactivation of E-cadherin interferes with the capacity of NHU cells to form stable calcium-mediated contacts and attenuates E-cadherin-mediated PI3-K/AKT induction, but enhances NHU cell proliferation by promoting the autocrine-driven EGFR/ERK pathway, which (via GSK3β phosphorylation and inactivation of the destruction complex) activates β-catenin-TCF signalling. Furthermore, if EGFR activity is blocked, then NHU cells are seen to be responsive to canonical Wnt signalling – either provided by addition of exogenous Wnt ligand or endogenously if NHU cells are cultured with palmitic acid to enable post-translational palmitylation of autocrine-produced Wnt ligands (Georgopoulos et al., 2014). This has revealed a complex, contextual interrelationship wherein the inherent capacity for self-repair is carried via at least 3 autocrine-regulated intracellular pathways that interact (“crosstalk”) through regulation of the activity/availability of key pathway components (minimally E-cadherin, EGFR, pERK, β-catenin, and pAkt), as summarized in Fig. 1.

The E-cadherin-defined adherens junction effectively acts as a master regulator through which urothelial cells individually “sense” and effect control over their neighbours to regulate population growth. The system is highly context-specific and self-regulating, with the local density of cells in the population and the propensity for cells to form cell:cell bonds affecting receptor availability and the downstream pathways that are activated. There is further regulation through specific (both positive and negative) feedback between the signalling pathways. Disruption of population homeostasis (eg by scratch-wounding a confluent contact-inhibited culture) results in cells of the population responding locally. These interactions are complex and, as we have shown previously when we revealed the PI3K pathway contribution (Georgopoulos et al., 2010), can be informed by insight from modelling.

The observation that NHU cell cultures are proliferative, but reversibly contact-inhibited at confluence was used as the basis to develop the agent-based computational framework called Epitheliome (Walker et al., 2004b). The non-deterministic nature of the Epitheliome was shown to predict regenerative behaviour in scratch-wounded monolayers (Walker et al., 2004a) and predicted an unidentified growth activation pathway present at low density in physiological [2 mM] calcium cultures. This observation led directly to the identification of the cell contact-mediated PI3-K/Akt signalling pathway described above (Georgopoulos et al., 2010).

The current study has been in part motivated by the need identified in our previous modelling work to accurately classify the behaviour of in vitro cell cultures under different experimental conditions and ultimately, carries the goal of automated extraction of rules and parameters to reliably inform computational models.

### 2.2 The state of the art in the modelling of biological tissues

Previously, we have used agent-based models (ABMs) in order to simulate NHU cells at the level of the individual cell, and make predictions about the emergent population-level behaviour under different culture conditions. Briefly, in an ABM, each individual real world entity (in this case, biological cell) is represented by an equivalent virtual entity, or “software agent” which performs simple behaviours and interacts with its local neighbours using pre-programmed rules. We have explored the contact-mediated effects on cell population growth (Walker et al., 2004b, Walker et al., 2010) and wound healing (Walker et al., 2004a, Walker and Southgate, 2013) using an agent-based representation of NHU cells in low and physiological calcium conditions.

These models included rules for cell migration, adhesion and proliferation according to the immediate neighbours and extracellular conditions of individual agents. These rules, as is the case for other ABMs of cellular systems, were derived from the observation of small numbers of individual cells in culture using time-lapse microscopy. Where such data were unavailable, rules were based on assumptions or hypotheses relating to how cells would reasonably be expected to behave under various conditions: the latter being drawn from both the literature and “domain knowledge” of researchers working with the experimental system.

Assumptions and approximations relating to the qualitative and quantitative nature of cell behaviour are thus inherent in ABMs (as with other modelling approaches), giving rise to epistemic uncertainty in the model predictions. Recent research effort has focused on the development of uncertainty analysis techniques in order to attempt to quantify the effects of epistemic and aleatory (stochastic-based) uncertainty in predictive models (Alden et al., 2013). Though robust, such methods have the disadvantage of being computationally intensive, requiring large numbers of simulations exploring a potentially large parameter space. However, the automated tracking and evolutionary analysis techniques described here offer an alternative approach to reducing the problem of uncertainty in models. If agent rules can be directly informed by statistically-sound and objective observations of cell behaviour extracted directly from microscopy image sets, this will increase confidence in the accuracy of model predictions and substantially reduce the search space of any subsequent exploration of model uncertainty.

### 2.3 Cell tracking and characterization

A range of cell tracking and characterization methods is described in the literature. Meijering et al. (Meijering et al., 2012) reviewed various computational approaches and quantitative measures for tracking and characterizing cells using time-lapse microscopy. Whilst exploring the requirements for tracking cells, it is clear that a number of stages, including pre-processing of the source images, appropriate tool selection and verification, need to be considered carefully to ensure that the approach adopted is optimal for the particular application under consideration. This is reflected by the range of other approaches presented in the literature: Srinivas and colleagues described a multimodal imaging approach to cell tracking using MRI, fluorescence, SPECT, PET and bioluminescence (Srinivas et al., 2013), and although effective, is resource intensive and time consuming to the point where it can become logistically prohibitive. More recent methods have been proposed which claim efficient tracking performances (such as (Seeto and Lipke, 2016), (Amat et al., 2015) and (Paintdakhi et al., 2016)). Other factors, such as the reliability of tracking cells as they leave and enter the field and depth of view are also of major consideration; Chatterjeea et al. proposed using matching and linking of bipartite graphs, which they claim does not require an explicit motion model, is highly scalable and can effectively handle the entry and exit of cells from the field of view (Chatterjee et al., 2013).

Having reviewed these existing techniques, there remains a strong motivation for developing bespoke tools that can be tailored to match the image quality and characteristics of the cell cultures under consideration. Such an approach also permits the tuning of tools to ensure that performance is adequate to facilitate analysis of cell cultures within a practical time scale. Finally, it is important that the results of this processing are compatible with other novel characterisation and classification approaches, such as evolutionary algorithms.

### 2.4 An introduction to evolutionary algorithms

The evolutionary analysis techniques employed in this work, Evolutionary Algorithms (EAs) (Chiong et al., 2012) are members of the artificial intelligence family, or more precisely computational intelligence, as they depend on a form of learning inspired by Darwinian evolution. They are in effect a number (or *population*) of candidate solutions (*individuals*) to a classification problem that are repeatedly refined (or *evolved*) over a number of iterations (*generations*) until a suitably accurate classifier algorithm is obtained, or the computational resources have been exhausted. The procedure for finding a classifier, for example to discriminate one cell culture from another, can be summarized as follows: A population of individuals (candidate solutions) is randomly initialized. The effectiveness or fitness of each individual to correctly classify data previously obtained from the cell cultures is determined using a fitness function. The fittest individual (the one with the highest fitness score determined by the fitness function) is retained and the others discarded. Copies (or *clones*) of this fittest individual are then generated and subtly modified (or mutated) to form a new population of individuals. The fitness of this new population of individuals is then evaluated in the same way using the fitness function, and the process is repeated over a number of generations until a sufficiently fit classifier is obtained, or the number of predetermined generations has been reached. Many different types of evolutionary algorithm have been developed which specify, not only the characteristics of the evolutionary process, but also the representation of the individual candidate solutions.

## 3 Methods

The automated analysis of cell motion comprises the following sequence of analysis: capturing images of cells in culture at regular intervals by videomicroscopy, tracking cells within the video on a frame-by-frame basis using custom-written software followed by characterization of cell movement through the extraction of specifically designed features. Each of these stages is considered in further detail in the following sections.

### 3.1 Cell culture and videomicroscopy

**Fig. 2:**
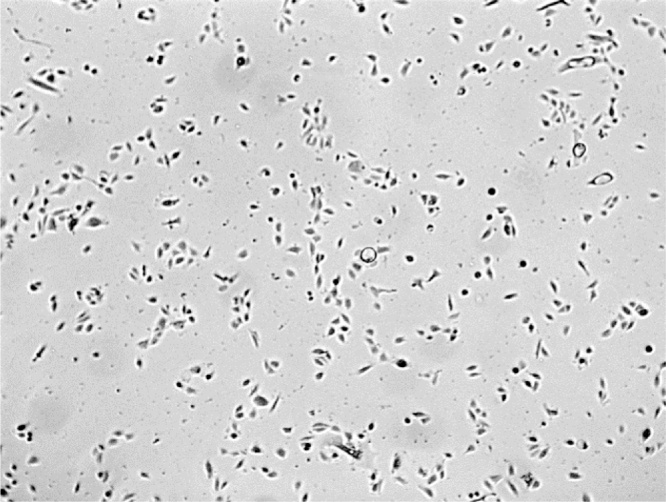
Sample frame from time-lapse video of NHU cells in culture.

Normal human urothelial (NHU) cells were established in culture as finite (non-immortalized) cell lines and maintained as detailed elsewhere (Southgate et al., 1994). Cultures were seeded in 12 well plates and exposed to 100 μM PPADs (pyridoxalphosphate-6-azophenyl-2′,4′-disulphonic acid) or 0.1% DMSO (as vehicle control) for 10 min, before addition of 0, 10 or 50 μM ATP in replicates of four. Cultures were observed using ×4 objective by differential interference contrast videomicroscopy (Olympus IX81 microscope) in an environmental chamber with an automated mechanical stage. Time-lapse videos were compiled from individual images captured digitally every 5 min over a 24 h time period. A sample frame from one such video is illustrated in Fig. 2.

### 3.2 Cell tracking

Custom-written software was developed to undertake automated cell tracking using the OpenCV computer vision programming library (Bradski, 2000). In order to track the relative movement of cells within a video, each frame undergoes processing to identify the likely locations of cells. This process takes the raw videos as an input, performs common pre-processing to each frame, and then either tries to identify the likely location of cells, or track the location of previously located cells. These steps are explained in further detail below, as previously reported by the authors (Zhang et al., 2015).

Each video frame initially undergoes Gaussian blurring to remove noise, followed by simple thresholding against a predetermined fixed value, resulting in a binary image separating the foreground and background (i.e. the cells from the frame background). Further processing (in the form of a distance transform) is then applied to this binary image, resulting in frames where the centres of large cells (or groups of cells) are assigned a large value, the edge of cells a lower value, and the background a value of 0. In order to efficiently estimate the locations of the centres of cells, the local maxima of the distance images are computed. Local maxima are then selected from the highest to lowest scoring, with a small area around each selected maxima being filtered out to reduce the number of selections made within the body of a cell. The (x,y) coordinates of the selected maxima are then used as estimations of the locations of cells within the frame.

To estimate the location of a cell within a frame, given the location within the previous frame, the distance image around the previous cell location is first multiplied by a simple Gaussian filter. The maximal pixel value in this region can then be used to estimate the new cell location. This approach, although simplistic, is demonstrated to be effective. The usage of the distance image promotes matches with the centre of cells, whilst the application of the Gaussian filter means that matches are preferred that are close to the original location of the cell.

Although the process for tracking cells works well, it is unable to consistently identify and track cell locations for the duration of the videos. In order to detect as many cells as possible, a large number of potential cell locations are initially calculated, with many of these quickly converging to the same locations. Similarly, the cell tracking process can occasionally fail to track the location of cells within frames, meaning that if cell detection were only to be performed on the first frame of the video then many cells would not be tracked in the latter parts of the video. These difficulties associated with tracking cells can be due to cell proliferation (giving rise to new cells), cell death (the loss of cells), and cells moving in and/or out of the field of view.

**Fig. 3:**
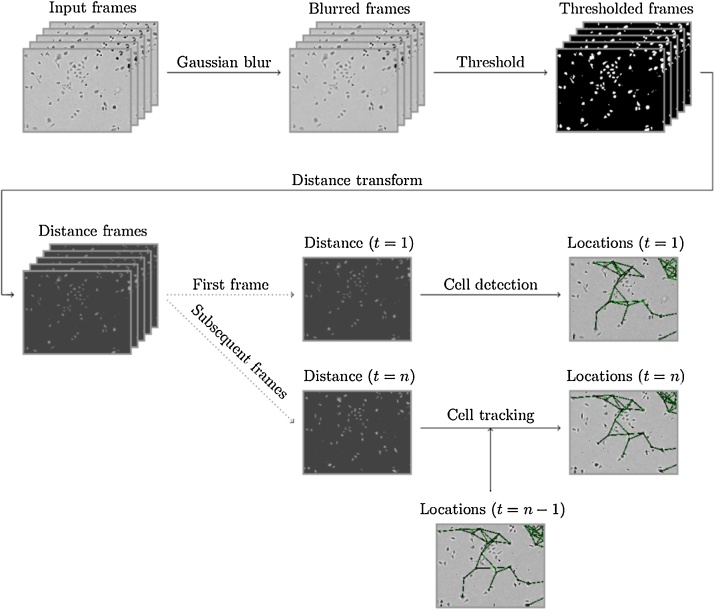
The process used for detecting cell locations within a video, and tracking of detected cells between video frames.

In order to cope with these issues, an approach was adopted where duplicated cell locations are removed from the tracking process and cell detection is performed at regular intervals to find new candidate locations. This approach is found to be effective and results in location data for a sufficient number of cells over the duration of the video to adequately describe the cell population. The entire cell tracking process is summarized in Fig. 3.

### 3.3 Feature extraction

**Fig. 4:**
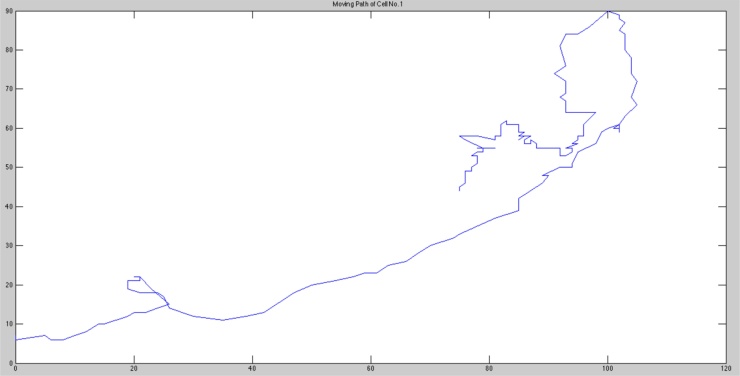
Example tracking of a single cell over a 24-h video, sampled every 5 min. Tracking began when the cell was right of centre, and continues until it approaches the lower left hand side of the reference frame.

Once the location of individual cells has been identified for each frame of the video in the form of (x,y) coordinate pairs, it is possible to extract features with the aim of describing the cell population behaviour. This was undertaken using the MATLAB programming environment and to illustrate the processing applied, a single cell from a video analysed is taken as an example to demonstrate how features of interest are calculated. The selected cell was tracked from a video of NHU cell culture with 50 μM exogenous ATP. As this cell was successfully tracked from the beginning to the end of the video, its path can be shown graphically as depicted in Fig. 4. It is interesting to note the significant change in cell behaviour from the start of tracking (near the centre of the graph) to end of tracking (when the cell leaves the lower left hand side of the reference frame); initially, the cell’s course is erratic, but subsequently stabilizes. The extraction of other features from the tracking data is anticipated to help us relate such changes in behaviour to factors in the environment, such as interactions with other cells, including intercellular adhesion or subsequent separation.

#### 3.3.1 Choice of features

The choice of features to extract from the videos was made on the basis of characteristics previously associated with cell behaviour and the computational feasibility of their extraction. Features can broadly be considered in two groups: (i) those that describe a cell’s behaviour over the entire time it was successfully tracked, and (ii) in terms of behaviour delimited by interaction, or more specifically, contact with other cells – described here as either *in-contact* or *post-contact*. The number of cells sharing the contact, or *clump size,* is also of interest. From visual inspection of the videos and for the purpose of this investigation, a clump has been defined as a group of five or more cells. The features are summarized in Table 1; all dynamic features are expressed in units of pixels per frame.

Further explanation of how the features *Average Migration Speed* and *Average Angular Velocity* have been defined is given below.

#### 3.3.2 Average migration speed

**Fig. 5:**
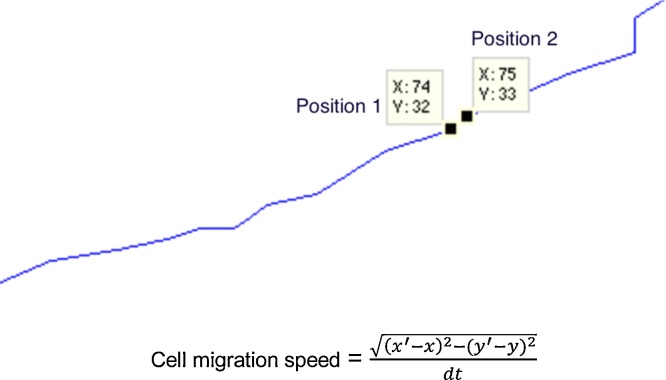
Example calculation of cell migration speed (pixels/frame).

The speed of an object is the rate of change of its position. In this case, the aim is to obtain the migration speed of a cell from a video, which can be determined by calculating the number of pixels travelled over a certain time interval. The time interval applicable in this context is that between two consecutive video frames, at a frame rate of one every 5 min. The migration speed is therefore simply obtained by calculating the Euclidean distance between the two pairs of coordinates for the cell between consecutive frames. This is shown graphically in Fig. 5 where the initial position of the cell is at coordinates (74,32) and in the subsequent frame, coordinates (75,33). Hence, the distance travelled by this cell over time *dt* (5 min), and subsequently, its speed, can be calculated. The migration speed of all cells tracked during the entire video was calculated in the same way.

#### 3.3.3 Average angular velocity (or migratory persistence)

In cell migration, persistence is one of the features in which biologists are most interested and can be described as the tendency of cells to change direction. Hence, obtaining the direction of travel of the cell in each frame of the video is essential for calculating migration persistence.

**Fig. 6:**
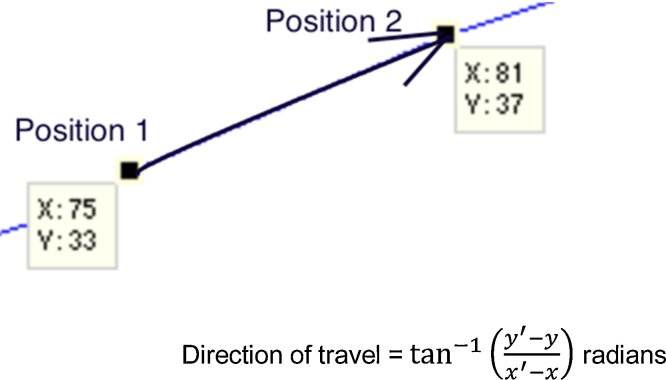
Direction of travel of cell migration.

**Fig. 7:**
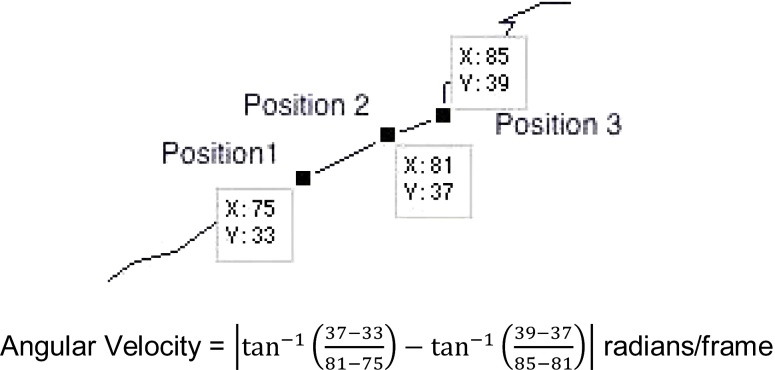
Example calculation of cell migration persistence over two consecutive frames.

Fig. 6 shows how the angle of the vector formed from the coordinates of the cell in consecutive frames of the video can be used to determine the direction of travel. Angular Velocity is defined here as the rate of change of the direction of travel of a cell over subsequent frames. Fig. 7 illustrates an example calculation over two consecutive frames.

### 3.4 Cell culture classification

The aim of extracting features such as those described in Section 3.3 is to permit characterization and, hence, classification of a cell culture. This can be achieved to some degree by simply observing the differences in measurements obtained by extracting these features from tracking data obtained from the respective cell videos. However, there are situations in which such a simple approach is insufficient to provide a full understanding of the differences between cell cultures (eg following drug treatment). The relationship between the features defined in Table 1 is complex and not well understood. In such situations, conventional, statistically-based classifiers do not always provide the best results and for this reason a computational intelligence approach was applied, in this case an evolutionary algorithm. Such approaches also have the advantage of being able to provide an insight to the characteristics of the cell culture that leads to this classification.

#### 3.4.1 Application of evolutionary algorithms

**Fig. 8:**
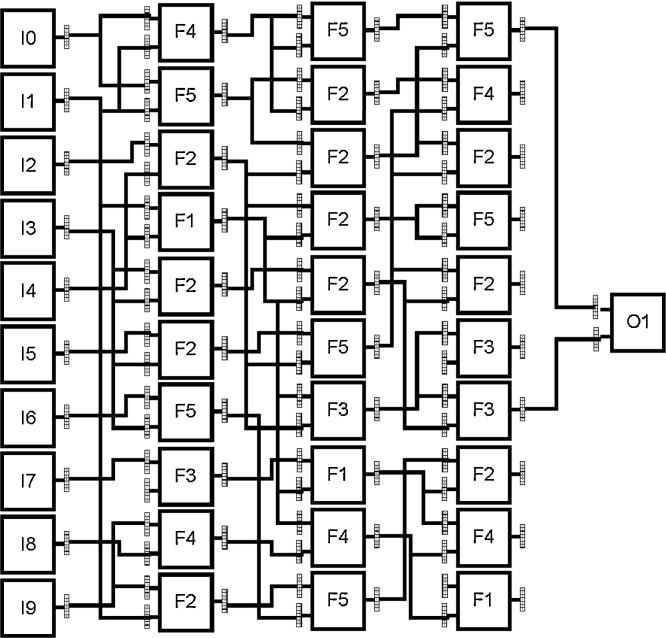
Example CGP network with 10 inputs I0-I9, three columns of processing nodes and one output O1.

For the work described here, Cartesian Genetic Programming (CGP) (Miller and Turner, 2015) was used: a form of Genetic Programming that comprises a fixed, non-cyclic directed graph, as shown in Fig. 8. This graph is effectively a network of processing elements that can be reconfigured during the evolutionary process in two fundamental ways: by selecting the function for each node from a predefined list, and specifying to which other nodes each input and output of the node are connected. In the example shown in Fig. 8, the features 1–10 extracted in Table 1 are presented to inputs I0-I9. These are then processed by the following three columns of nodes, each executing an arithmetic function taken from a set F1...F5, typically primitive arithmetic operations such as illustrated in Table 2; the result of the network is presented at output O1.

There are two ways in which CGP provides an advantage over other classification techniques. First of all, for highly non-linear complex data sets, such as those found in measurements of dynamic behaviours, CGP has been shown to evolve high performance classifiers (Lones et al., 2014). Secondly, unlike other machine learning techniques, once a high performing classifier has been evolved, a mathematical expression defining this classifier can be easily obtained by decoding the resulting CGP network. As previously mentioned, this can provide valuable insight into those features obtained from the dynamics of the cell culture that play a defining role in its classification.

## 4 Results

In total 24 time-lapse videos were generated from six classes of NHU cell cultures comprising combinations of control cultures, with and without ATP and PPADS, as detailed in Table 3, and were analysed using the methods described above in Section 3.

### 4.1 Evaluation of results

An initial evaluation of the results obtained considered a subset of three classes of NHU cell cultures: (i) a control culture with no ATP; (ii) a culture with 10 μM ATP; and (iii) a culture with 50 μM ATP. The average cell migration speeds and average angular velocity for each video were calculated and are presented in Table 4.

**Fig. 9:**
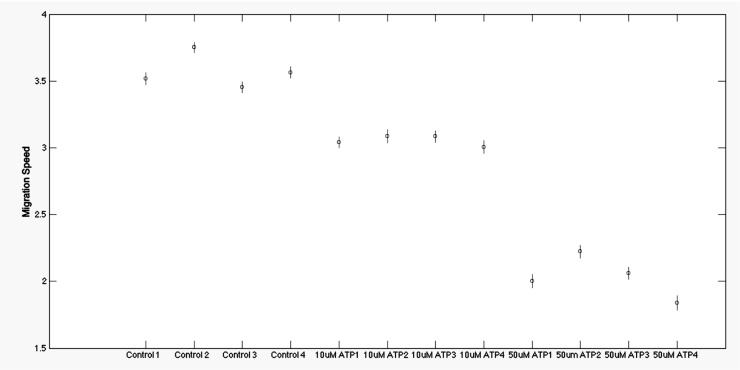
Automated calculation of cell migration persistence. Average migration speeds are shown in F-distribution form for Control, 10 μM ATP and 50 μM ATP Videos: small circles mark the mean of the group and the bars the 95% confidence interval.

**Fig. 10:**
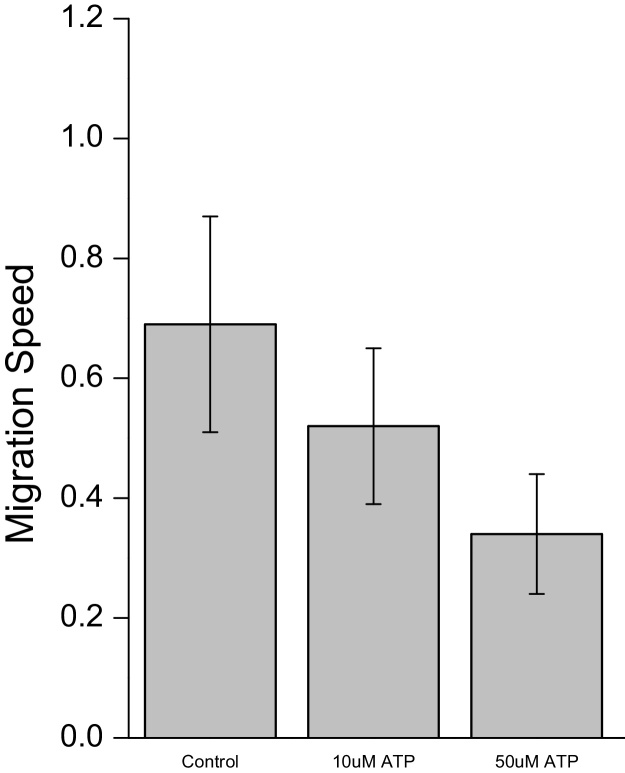
Calculation of cell migration persistence using manual tracking.

By applying analysis of variance (ANOVA), it can be seen in Fig. 9 that the separation between the three classes for migration speed was statistically significant. Verification of these results was confirmed by comparing with manual tracking of 15 random cells for each experimental condition as shown in Fig. 10. Similarly, results for angular velocity, shown in Fig. 10, also demonstrated good separation between the three sets of culture conditions.

### 4.2 Full feature set results

**Fig. 11:**
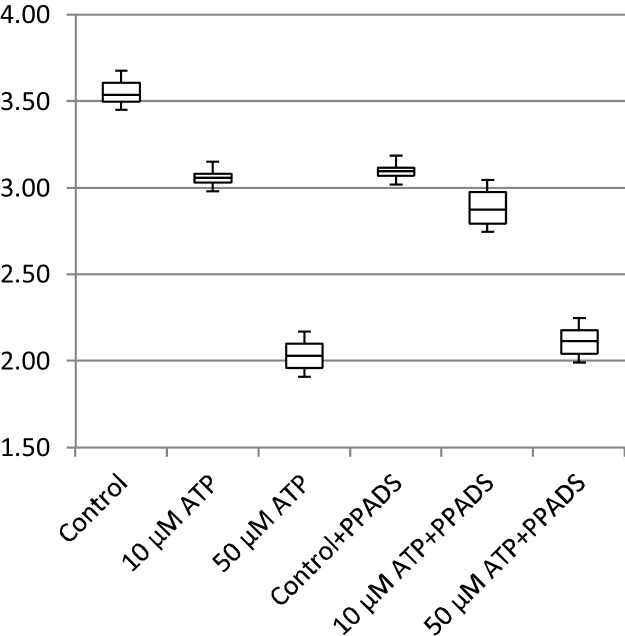
Average migration speed (pixels per frame).

**Fig. 12:**
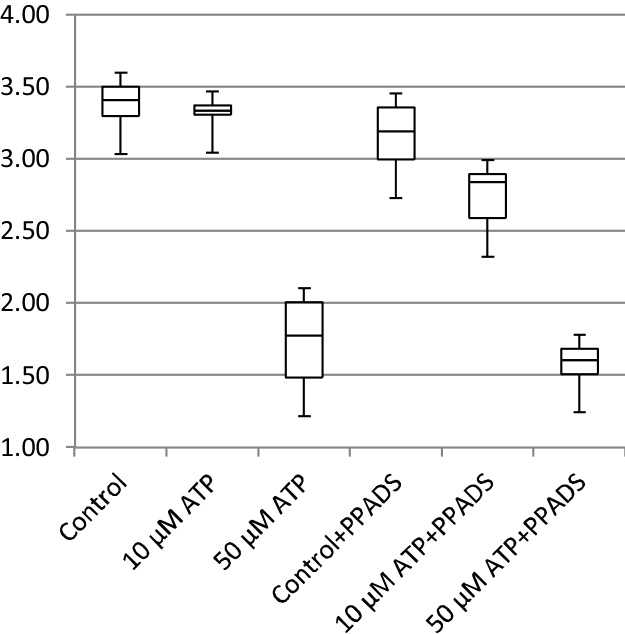
Post-contact migration speed (pixels per frame).

**Fig. 13:**
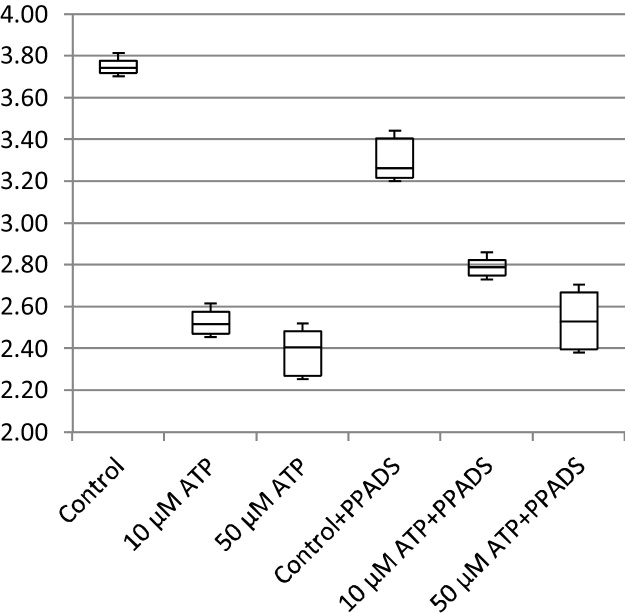
In-contact migration speed (pixels per frame).

**Fig. 14:**
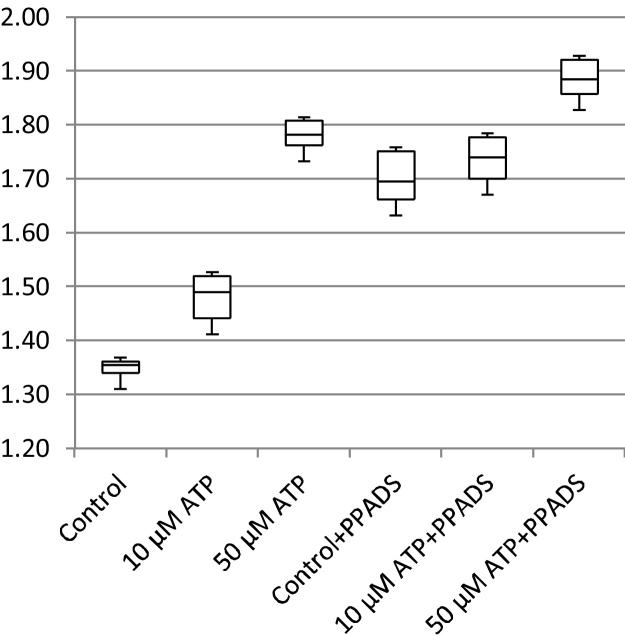
Average angular velocity (migratory persistence) (radians per frame).

**Fig. 15:**
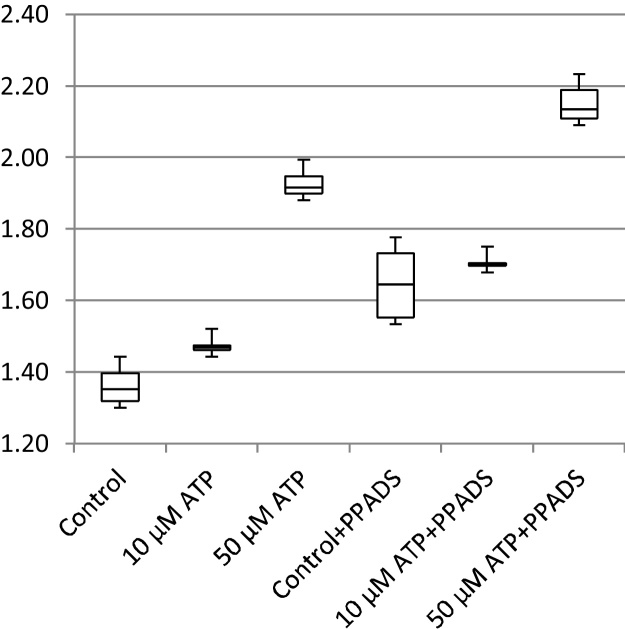
Post-Contact Angular Velocity (radians per frame).

**Fig. 16:**
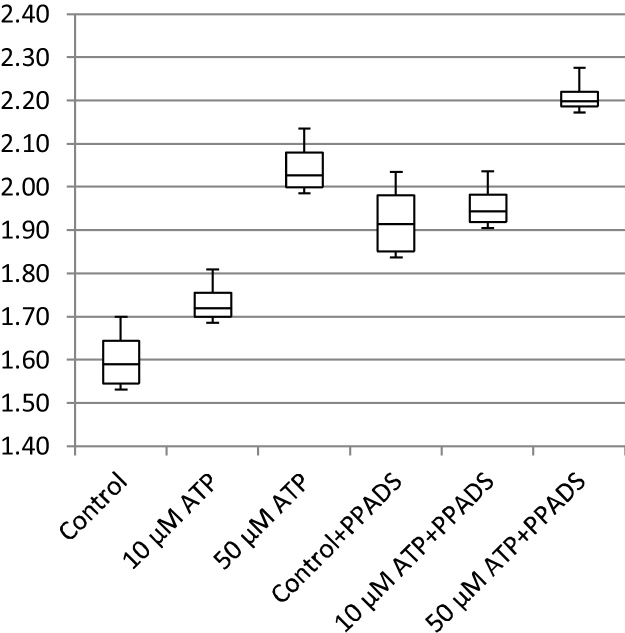
In-Contact Angular Velocity (radians per frame).

**Fig. 17:**
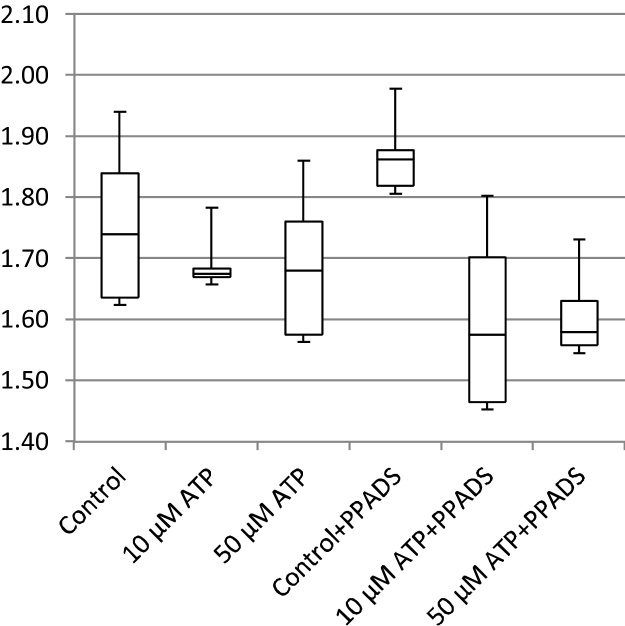
Cohesivity (average number of contacts per cell).

**Fig. 18:**
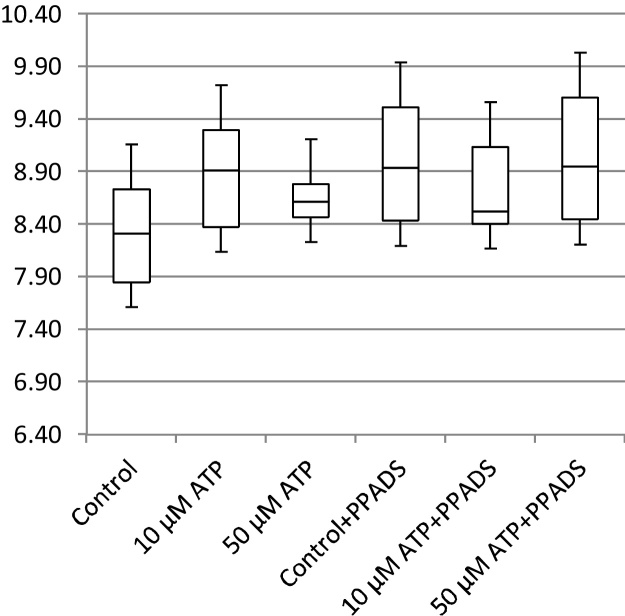
Average cell clump size (number of cells).

**Fig. 19:**
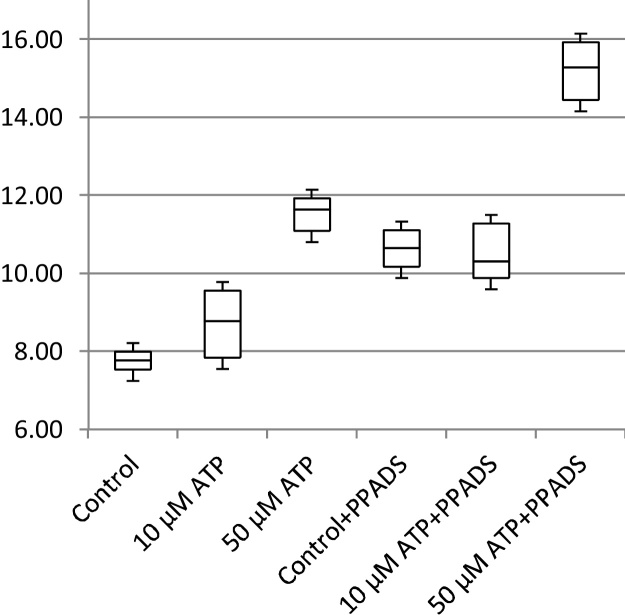
Average contact duration (in frames).

**Fig. 20:**
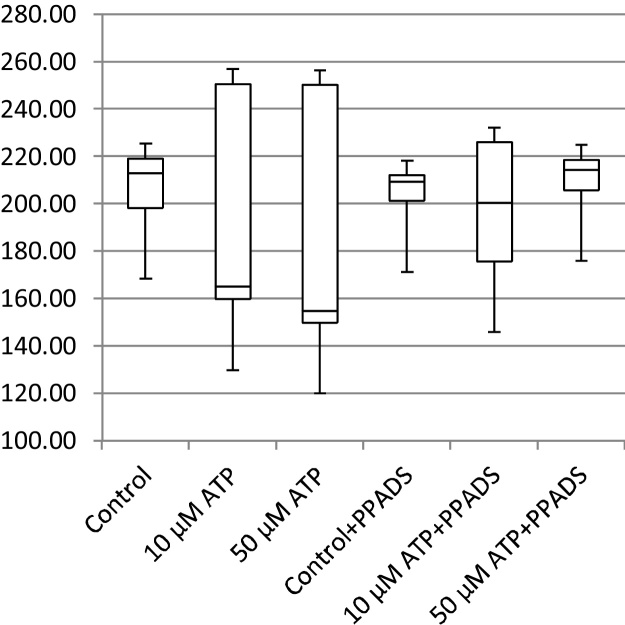
Cell count.

In addition to cell migration speeds and migratory persistence, we are particularly interested in the nature of the contact between cells. This relates to the physical extent of the contact that forms between them and to what extent interacting cells make transient or more sustained contacts. The features, previously defined in Table 1, that characterize the behaviour of cells whilst in contact and post-contact throughout the videos are shown in Fig. 11, Fig. 12, Fig. 13, Fig. 14, Fig. 15, Fig. 16, Fig. 17, Fig. 18, Fig. 19, Fig. 20.

### 4.3 Application of evolutionary algorithms

It can be seen in the results presented in Section 4.2 that cell cultures containing PPADS cannot be clearly distinguished from those cultures without PPADS in every case. As the relationship between the features is complex and not well understood, an evolutionary algorithm was used in an attempt to generate an effective classifier and provide some insight on the role of the features in the classification. A CGP network was used, described by the parameters listed in Table 5. In total, five runs of 10 separate experiments were undertaken, achieving an average accuracy of 91.4% on independent test set data.

**Fig. 21:**
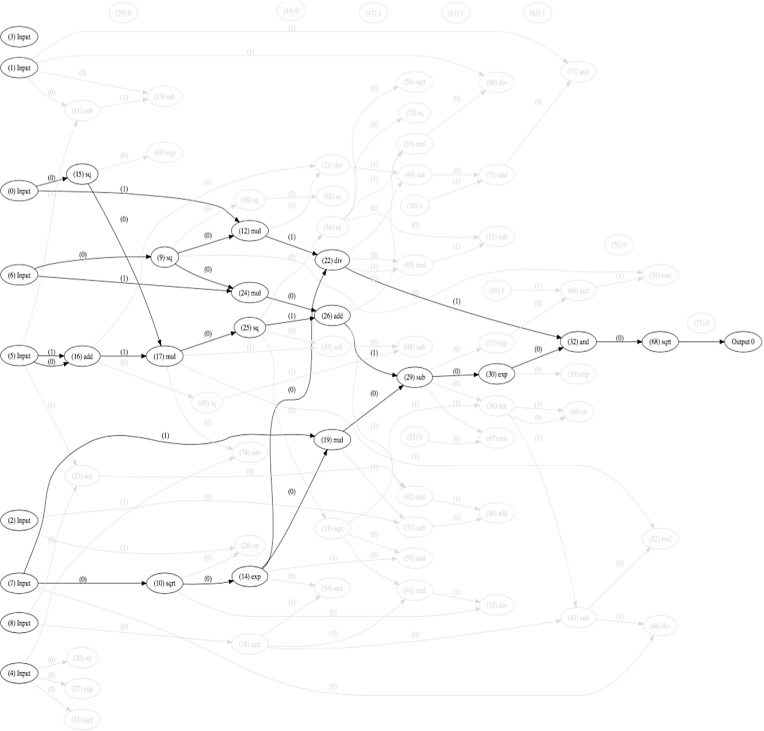
Evolved CGP network solution that achieved 94% classification accuracy between cell cultures with and without PPADS. Inputs (0)–(9) represent features 1–10 as defined in Table 1. The bold nodes and paths represent the active network (whilst greyed-out nodes and paths are not used, but may have been instrumental in the evolution of the classifier in previous generations).

An evolved CGP network that successfully classifies cell cultures with and without PPADS to an accuracy of 94% is shown in Fig. 21. The features defined in Table 1 were presented to the inputs of the network and the value obtained at the output is used to obtain the classification result.

The added advantage of applying evolutionary algorithms in this way is that the evolved network can easily be described as a conventional mathematical expression. This provides valuable insight into the features most significant in the classification process and their relationship. For example, in the example provided in Fig. 21, it can be seen that this particular classifier is dependent on inputs: (0), (5), (6) and (7), which, with reference to Table 1, can be equated to features: average migration speed, in-contact angular velocity, cohesivity and average cell clump size, respectively.

## 5 Discussion and conclusions

This paper has described a novel approach to the characterization and classification of replicate cell cultures through the application of cell tracking to time-lapse videos. A number of features have been proposed that provide a means of describing cell behaviour in terms of migration speed and migration persistence, both while in contact with other cells and post-contact. This provides a unique opportunity to infer behaviour with respect to cell contact in a fully automated way. It has also been demonstrated how evolutionary algorithms can be used to successfully classify cell cultures even when this is not clearly indicated by considering the extracted features alone.

In the specific example of NHU cells studied here, we previously reported that in scratch assays, where the repair of damage inflicted on a confluent population of cells is monitored to the closure of the wound, the effect of exogenous ATP was to enhance wound repair. In support of these observations, inhibition of ATP breakdown was shown to enhance the rate of repair, whereas PPADs, a selective antagonist of the ATP-activated P2X receptor, was inhibitory (Shabir et al., 2013). These observations led us to predict that ATP had a positive effect on cell migration and it was unexpected here that in sparse cell cultures, the effects of exogenous ATP was to reduce migration speed. These observations, along with the equivocal effect of the P2X antagonist PPADS, suggests that the response of NHU cells to ATP may be more context dependent than hitherto thought and other urothelial-expressed ATP-modulated receptors, such the P2Y G protein-coupled receptors (Shabir et al., 2013) may be relevant. The novel method of analysis presented in this paper provides the means by which predominant affected parameters and the mechanisms responsible can be fully examined in an automated and objective way.

## Figures and Tables

**Table 1:** Summary of features extracted from cell culture videos.

Feature No.	Feature Name	Description
1	Average Migration Speed	Average speed of cells over the period of tracking
2	Post-Contact Migration Speed	Average migration speed of cells over three frames after leaving a clump of cells
3	In-Contact Migration Speed	Average migration speed of cells when in contact with five or more other cells (a *clump*)
4	Average Angular Velocity (or *Migratory Persistence*)	Rate of change in migration direction of cells between two video frames
5	Post-Contact Angular Velocity	Rate of change in migration direction of cells after leaving a clump
6	In-Contact Angular Velocity	Rate of change in migration direction of cells when in a clump
7	Cohesivity	Average number of contacts per cell
8	Average Cell Clump Size	Average size of clump in number of cells
9	Average Contact Duration	Average duration of cell contact with clump
10	Cell Count	Difference between the maximum number of cells tracked during the video from the number tracked at the beginning

**Table 2:** Example function set providing a lookup table for functions F1 to F5 used in the network illustrated in Fig. 8.

Function	Arithmetic Operation
F1	+
F2	–
F3	*
F4	/
F5	mean

**Table 3:** Automated average migration speed and average angular velocity values for a control culture with no ATP, a culture with 10 μM ATP and a culture with 50 μM ATP.

Cell Culture Video	Cell Culture Description
1–4	Control
5–8	10 μM ATP
9–12	50 μM ATP
13–16	Control + PPADS
17–20	10 μM ATP + PPADS
21–24	50 μM ATP + PPADS

**Table 4:** Automated average migration speed and average angular velocity values for a control culture with no ATP, a culture with 10 μM ATP and a culture with 50 μM ATP.

Cell Culture Video	Average Migration Speed (pixels/frame)	Average Angular Velocity (rads/frame)
Control1	3.52	1.31
Control2	3.75	1.35
Control3	3.45	1.37
Control4	3.56	1.36
10 μM ATP1	3.04	1.52
10 μM ATP2	3.09	1.39
10 μM ATP3	3.08	1.52
10 μM ATP4	3.01	1.46
50 μM ATP1	2.00	1.79
50 μM ATP2	2.22	1.74
50 μM ATP3	2.06	1.77
50 μM ATP4	1.83	1.85

**Table 5:** Parameters specifying the CGP network used to evolve classifiers to discriminate between cell cultures with and without PPADS.

Parameter	Value
Number of inputs	9
Number of outputs	1
Number of columns	70
Number of rows	1
Mutation rate	1.0%
Population size	10
Function set	
Arithmetic operators:	+-* / SQR SQRT CUBE
Constants:	0 1
Logical operators:	AND OR NAND NOR NOT
Number of generations	10,000
